# A Twenty-Year Follow-Up of Adults with Ebstein Anomaly with Special Focus on Supraventricular Arrhythmias, Supraventricular Arrhythmias and Effectiveness of Catheter Ablation in 20-Year Follow-Up of Adults with Ebstein Anomaly

**DOI:** 10.3390/jcm13072039

**Published:** 2024-04-01

**Authors:** Sonia Alicja Nartowicz, Aleksandra Ciepłucha, Michał Waśniewski, Izabela Miechowicz, Justyna Rajewska-Tabor, Agnieszka Bartczak-Rutkowska, Maciej Lesiak, Małgorzata Pyda, Olga Trojnarska

**Affiliations:** 1 1st Department of Cardiology, Poznan University of Medical Sciences, 60-806 Poznań, Poland; aleksandra.cieplucha@skpp.edu.pl (A.C.); michal.wasniewski@skpp.edu.pl (M.W.); iza@ump.edu.pl (I.M.); justyna.rajewska@skpp.edu.pl (J.R.-T.); maciej.lesiak@skpp.edu.pl (M.L.); malgorzata.pyda@skpp.edu.pl (M.P.); olga.trojnarska@skpp.edu.pl (O.T.); 2 Department of Computer Science and Statistics, Medical University of Poznan, 60-806 Poznań, Poland

**Keywords:** Ebstein anomaly, catheter ablation, supraventricular arrhythmias, adult congenital heart defect, outcome

## Abstract

**Background**: Ebstein anomaly (EA) is a rare congenital heart disease characterized by the apical displacement of the tricuspid leaflets, creating an enlarged functional right atrium. Supraventricular arrhythmias (SVA) are common, and catheter ablation remains challenging. SVA is considered a risk factor for sudden cardiac death in this population. Still, there are very few real-life data on the impact of SVA treated invasively or conservatively on a patient’s prognosis. We aimed to analyze the incidence of SVA in adults with EA, evaluate the effectiveness of catheter ablation, and analyze the impact of SVA and catheter ablation on survival in this population. **Methods and results**: 71 pts (median age 53 years; range 24–84 years) with EA were evaluated retrospectively from 1988 to 2020. Forty patients (56.3%) had SVA, and eighteen of them (45.0%) required at least one catheter ablation (35 procedures in total). Indications for ablation were mostly intra-atrial reentrant tachycardia (IART) and atrioventricular reentrant tachycardia (AVRT) (14 pts [77.8% and 9 pts [50.0%], respectively. IART and AVRT coexisted in nine pts. One patient suffered from persistent atrial fibrillation. Procedural effectiveness was reported in 28 (80%) cases; over a longer follow-up (mean 12.6 ± 5.4 years), only eight (44.4%) patients were completely free from SVA after the first ablation. In total, 10 patients (14%) died due to cardiovascular events. There was no difference in survival between patients with or without SVA (*p* = 0.9) and between ablated and non-ablated EA individuals (*p* = 0.89). **Conclusions**: Supraventricular arrhythmia is frequent in adults with Ebstein anomaly. Patients often require more than one catheter ablation but eventually become free from arrhythmias. The imaging parameters assessed by echocardiography or cardiac magnetic resonance do not seem to be associated with ablation outcomes. The impact of supraventricular arrhythmia itself or treatment with radiofrequency ablation is questionable and should be thoroughly investigated in this population.

## 1. Introduction

Ebstein anomaly (EA) is a rare congenital heart disease (CHD) accounting for less than 1% of congenital heart defects [1]. This malformation of the leaflets of tricuspid valve (TV) often leads to a significant regurgitation. As a consequence, the right atrium and ventricle become enlarged, and this may result in unfavorable outcomes including supraventricular arrhythmias (SVA), heart failure, thromboembolic events, and sudden cardiac death (SCD) [2,3,4]. The most common type of SVA is atrioventricular reentry tachycardia (AVRT), but patients with EA are also at moderate risk intra-atrial reentrant tachycardia (IART) or focal atrial tachycardia [3].

Radiofrequency ablation (RFCA) is currently recommended in symptomatic EA patients as an alternative to antiarrhythmic drugs and/or electrical cardioversions [5]. However, the distorted anatomy of the right atrium in EA patients makes this procedure challenging and lowers the success rates compared to a structurally normal heart [6]. The anatomical abnormality comprises the displacement of the tricuspid septal and posterior leaflets toward the right ventricular “apex” while maintaining the hinge point of the anterior leaflet in the TV annulus [7]. Subsequent abnormal phenomena, including stretching of the chambers of the right heart, myocardial fibrosis, slower electric impulse conduction time through the myocardium, altogether, create an increasingly proarrhythmic environment. Additionally, the accessory conduction pathways originating from abnormalities in embryological development within the annulus and leaflets formation are described in more than 1/3 of patients with EA [8].

Due to the rarity of this anomaly, there is still a shortage of data on the effectiveness of RFCA in adults with Ebstein anomaly with SVA. When informing patients about the risks and benefits of invasive antiarrhythmic treatment, it is crucial to address the efficacy and potential long-term outcome. It is believed that in Ebstein patients, SVA is a risk factor of sudden cardiac death, mostly because of the rapid conduction of electric impulses from the atria to the ventricle. However, the impact of SVA on overall survival, not just SCD, in this population needs to be better established. Our study aims to determine the frequency of supraventricular arrhythmia in patients with Ebstein Anomaly, as well as to define its impact on the survival of this population, depending on the method of treatment of the arrhythmia and the severity of the defect, determined based on echocardiography and magnetic resonance imaging.

## 2. Method

### 2.1. Patient Population

We retrospectively analyzed 71 patients diagnosed with EA and followed up in our outpatient clinic between 1988 and 2021. Clinical characteristics were collected from the medical records, including coexistence of other congenital defects, surgical interventions, multiple standard 12-lead ECGs, and echocardiography of all patients. The data from 24-h Holter ECG monitoring and cardiac magnetic resonance were retrieved where available.

### 2.2. ECGs and Holter Ecg Monitoring

A standard 12-lead ECG was performed in all patients with the assessment of basic parameters, including the sinus/non-sinus rhythm, QRS complex duration, and the presence of a bundle branch block. All available ECG traces and ECG 24-h Holter records were re-evaluated with regard to the presence of SVA, including accessory pathways or atrial arrhythmias (focal atrial tachycardia, atrial flutter/intra-atrial reentrant tachycardia or atrial fibrillation). The classification of arrhythmia was based on the current ESC guidelines [5].

### 2.3. Cardiac Imaging

Echocardiography was performed with a VIVID 9 GE Medical System device, using a 2.5 Hz probe in 2D mode, in standard and modified views due to altered anatomy. All the echocardiographic measurements of the left and right heart were interpreted according to the current guidelines. In particular, the end-diastolic diameter of the RV proximal outflow tract (parasternal long axis view) exceeding 35 mm was considered a dilated RV [9]. The LV end-diastolic diameter exceeding 58 mm was considered dilated, and LV ejection fraction <52% was considered as decreased [9]. Echocardiographic classification of EA severity, called the Celermajer index (echoCel-ind), was performed as previously described [10]. The calculated values were used to define four grades of increasing severity of EA: echo1 (echoCel-ind < 0.5); echo2 (echoCel-ind 0.5 to 0.99); echo3 (echoCel-ind 1 to 1.49); echo4 (echoCel-ind > 1.5). We considered grades 1 and 2 to be a mild form of EA, whereas grades 3 and 4 were labeled severe. In ablated individuals, the echocardiographic parameters from the periprocedural examination were additionally analyzed to describe a potential relationship between the anatomy and the outcome of ablation.

Cardiac magnetic resonance was performed on a 1.5 Tesla scanner (Magnetom Avanto, Siemens, Munich, Germany) using a six-element matrix coil combined with a 2–4 element spinal coil. All images were ECG-gated and performed during patient breath-holds. During the standard protocol for EA, among other sequences, steady-state free precession imaging was used to assess the volume of the ventricles and atria. Standard 4-, 3-, and 2-chamber views with a stack of short-axis views and contiguous axial slices covering the heart from the RV outflow tract to the diaphragmatic surface of the right ventricle were used. The imaging parameters were as follows: TR/TE 2 R-R intervals/27 ms; the field of view, 380 × 260 mm; slice thickness, 8 mm without gap; and matrix size, 104 × 256.

The compartments of the right heart were as previously described [10]. The end-diastolic volume (EDV) and end-systolic volume (ESV) of the right atrium and ventricle were measured on axial planes. The left ventricle and atrium parameters were calculated in short-axis slices. The trabeculae and papillary muscles were included in the blood pool. All the measurements were carried out using dedicated software (QMass, Medis, Leiden, The Netherlands).

All the measurements of the left and right heart were interpreted according to the current guidelines on cardiac magnetic resonance [11]. In particular, normal RV end-diastolic volume indexed by BSA and RV ejection fraction were within the following ranges: for women, 48–112 mL/m^2^ and >51%; for men, 61–121 mL/m^2^ and >52%, respectively [11].

### 2.4. Electrophysiology Study and Radiofrequency Ablation

During the procedure, the patients were under local anesthesia; vascular access was obtained through the femoral vein. The 3-dimensional electro-anatomical mapping using the CARTO system (Biosense-Webster, Irvine, CA, USA; Johnson and Johnson, New Brunswick, NJ, USA) was implemented where applicable. Out of 35 procedures, 7 were performed before 2007. The procedure was considered effective if no SVA reappeared within 12 months of follow-up.

### 2.5. Statistical Analysis

The categorical variables are expressed as numbers and percentages and compared for statistical differences using the chi-square test. The continuous variables are expressed as median and range, or mean with one standard deviation, and compared with a two-tailed *t*-test. A *p*-value < 0.05 was considered statistically significant. To compare survival, we estimated Kaplan–Meier survival probabilities and used the log-rank test to determine significance (*p* < 0.05).

## 3. Results

### 3.1. Baseline Characteristics

**Figure 1:**
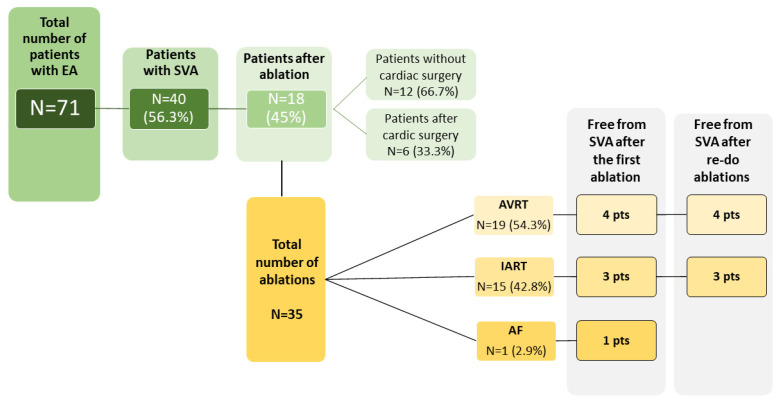
Characteristics of the study group in the context of the occurrence of supraventricular arrhythmia and invasive treatment.

We identified 71 patients (median age 53 years; range 24–84 years) with EA, from whom 40 patients (56.3%) had at least one episode of documented SVA. The baseline characteristics of the study group are summarized in Table 1 and Table 2 and Figure 1. The follow-up time at our center ranged between 1 and 28 years (median 7 years). In the ablated group, four patients had associated congenital heart defects—three patients had atrial septal defects and one had pulmonary stenosis. All the associated defects in ablated patients were surgically operated, of which two ASD closures were performed during the replacement of the tricuspid valve. Tricuspid valve surgery was performed in four patients; three of these patients underwent tricuspid valve replacement. Catheter ablation due to SVA was performed in 18 patients (25.4%); 15 patients (21.1%) received a pacemaker—in 10 (66.7%), the indication was advanced atrioventricular block, and in 5 (33.3%)—sinus node dysfunction. The vast majority of the patients [56 (78.9%)] were asymptomatic on a daily basis, functional New York Heart Association (NYHA) class II was present in 13 (18.3%) individuals, NYHA III—in two patients (2,8%). During the follow-up, there were 10 deaths (14.1%), all of them attributed to the cardiovascular event (heart failure in five cases, sudden cardiac death in two, thromboembolic event in two, and coronary artery disease in one patient).

### 3.2. Echocardiography

All patients underwent an echocardiography exam. The basic parameters of the whole group are shown in Table 3. In the entire study population, a normal dimension of the RV (<35 mm) assessed in the parasternal long axis view was found only in three (4.2%) patients. The end-diastolic dimeter and ejection fraction of the LV were within the normal range in all subjects. Anatomical classification of the defect was based on the Celermajer index, with 30 (42.3%) out of 71 and 7 (38.9%) out of 18 ablated patients labeled as severe defect (echo grade 3 and 4). There was no statistical difference in terms of echocardiographic parameters between ablated vs. non-ablated patients (LV diameter 37.4 ± 5.9 vs. 40.0 ± 4.7 mm, *p* = 0.06; RV diameter 55.5 ± 16.5 vs. 55.3 ± 13.9 mm, *p* = 0.9; RV/LV ratio 1.5 ± 0.5 vs. 1.4 ± 0.4, *p* = 0.4; Cel index 0.9 ± 0.3 vs. 0.96 ± 0.6, *p* = 0.9).

### 3.3. Cardiac Magnetic Resonance

Cardiac magnetic resonance was performed in 45 (63.4%) patients, including 10 with a history of ablation. Fifteen (33.3%) patients had left ventricular ejection fraction below 57%. The mean fRV ejection fraction was 40.3 ± 12.9% and was below the normal range in 30 (73.3%) subjects. The end-diastolic volume of fRV was increased in 20 (44.4%) subjects. Severe EA assessed with the Celermajer index (cmr grade of 3 or 4) was found in six (8.5%) patients.

In the subgroup of patients undergoing ablation, no patient had increased LVEDV and three (30%) patients had decreased LVEF. The end-diastolic volume of fRV was increased in 6 of 10 (60%) individuals. The mean fRV ejection fraction was 40.2 ± 11.6%, with decreased contractility reported in 8 of 10 (80%) patients. All subjects with a severe form of EA as assessed by CMR needed the radiofrequency ablation. Late gadolinium enhancement was present in 6 of 10 (60%) ablated patients, whereas in the whole group undergoing CMR, it was found in 17 of 45 patients. Late gadolinium enhancement within the functional right atrium (aRV + RA) was found in 5 of 10 (50%) ablated patients. Only 1 person of 10 (10%) had LGE in fRV and none in LV, whereas in the whole group, 9 (20%) patients had LGE in aRV + RA and 9 (20%) in fRV. No relevant relationship between CMR parameters and the need for ablation was found (Table 4).

### 3.4. Electrophysiology Study and Radiofrequency Ablation

All of the RFCAs were performed between 2001 and 2020. The total number of ablations was 35 with the median age of 34.5 years (range 11–67), (Table 2). Of the 18 ablated patients, at the time of intervention, sinus rhythm was present in 17 cases; one patient suffered from persistent atrial fibrillation (diagnosed at the age of 57). Intra-atrial reentrant tachycardia was the indication for intervention in 14 pts (77.8%); accessory pathways in nine (50%) patients. Both IART and AVRT co-existed in six individuals. In one patient, ablation was performed due to atrial fibrillation. Out of 18 patients treated invasively, 8 patients (44.4%) were completely free from SVA after the first ablation. In five (27.8%) patients, we observed the re-occurrence of arrhythmia within first 12 months. However, over a longer period of post-procedural follow-up (mean 12.6 ± 5.4 years), the number of reinterventions due to recurrent SVA increased. Most patients requiring reintervention were those with the diagnosis of AVRT, even though the short-term effectiveness of RFCA was 88%. In patients with IART, the first ablation was effective in 75% of cases. There were no major complications during the RFCA. Our analysis did not find any significant differences in the anatomical variables assessed by echocardiography or CMR between patients who required and did not require ablation (Table 3 and Table 4). However, we did not perform a separate analysis for patients after cardiac surgery due to the small number of operated patients (*n* = 6)

**Figure 2:**
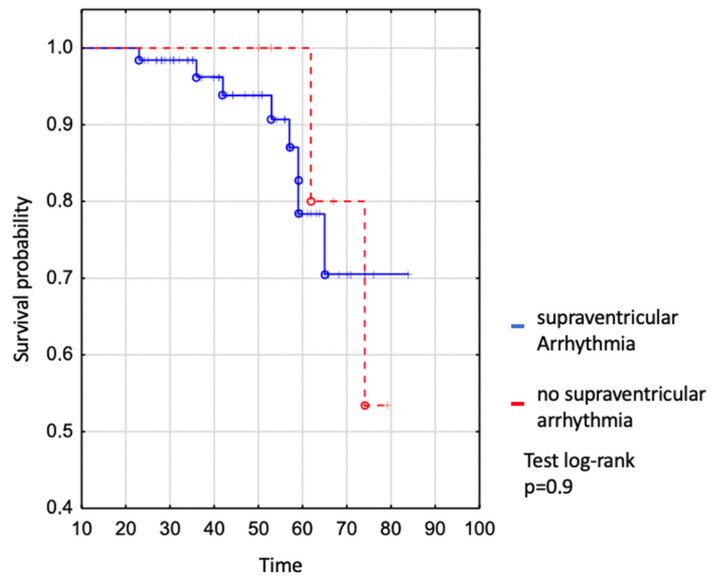
Kaplan–Meier curve: comparison of survival between EA patients with and without supraventricular arrhythmia.

We assessed survival in the whole EA patient group where no difference was shown between patients with and without supraventricular arrhythmia (Figure 2). The survival of patients with SVA was 94% to age 50 years, 78% to age 60 years, and 71% to age 70 years vs. 100% to age 50 years, 100% to age 60 years, and 80% to age 70 in those without SVT (log-rank *p* = 0.9), (Figure 2).

**Figure 3:**
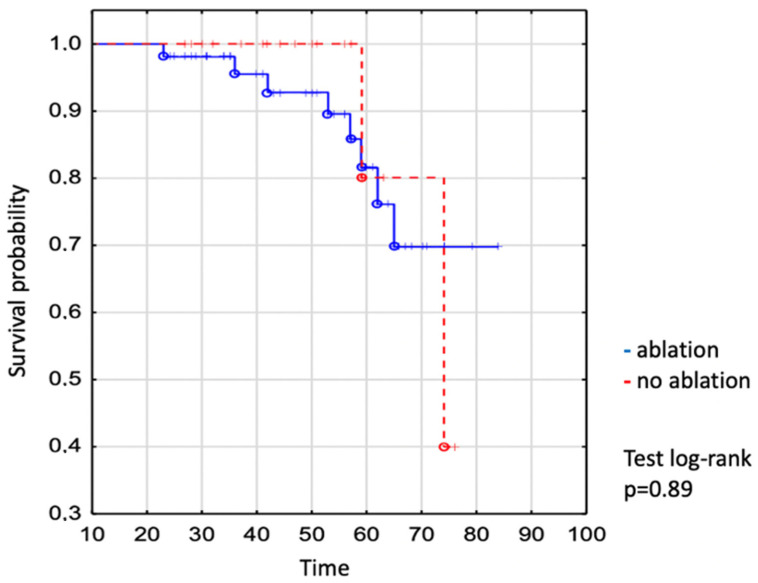
Kaplan–Meier curve: comparison of survival between EA ablated and non-ablated patients. The dot expresses the exact number of patients at the time.

We demonstrated no significant difference in the survival of individuals who required vs. did not require the intervention (Figure 3). The survival of patients undergoing catheter ablation was 93% to age 50 years, 82% to age 60 years, and 70% to age 70 years vs. 100% to age 50 years, 80% to age 60 years, and 80% to age 70 years in not-ablated patients (log-rank *p* = 0.89), (Figure 3).

**Figure 4:**
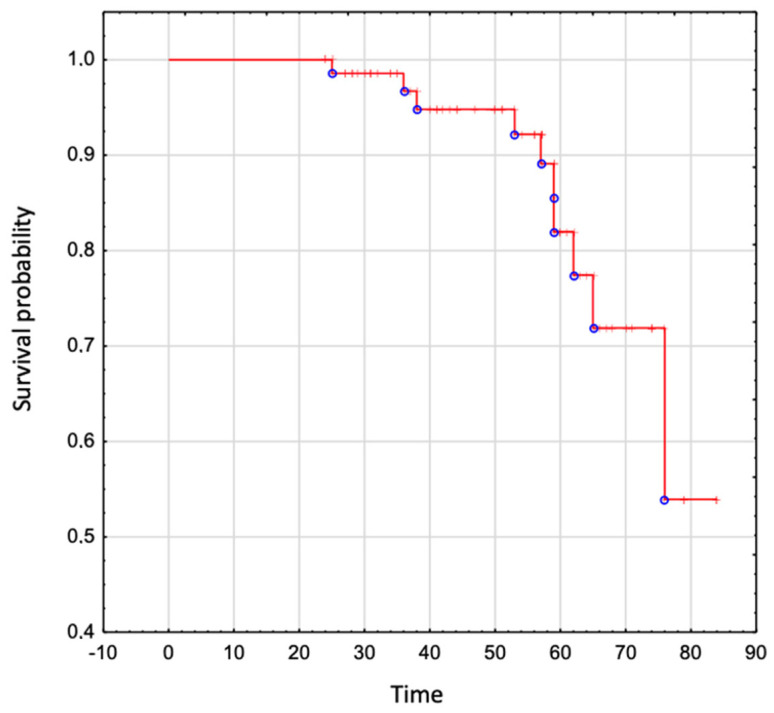
The overall survival in patients with Ebstein Anomaly.

The overall survival in the whole analyzed group was 95% to age 50 years, 82% to age 60 years, 72% to age 70 years, and 54% to age 80 years (Figure 4).

## 4. Discussion

In our study, the population size was probably too small to accurately determine the impact of supraventricular arrhythmia and its treatment on survival in the population of patients with Ebstein’s anomaly. Patients with EA are at the highest risk of developing supraventricular arrhythmia among the population with congenital heart disease [3]. Our observations show that more than half of patients develop supraventricular arrhythmias, mostly intra-atrial reentrant tachycardia and atrioventricular reentrant tachycardia, with 45% of them requiring a radiofrequency ablation. These data are consistent with previous reports in which interventional management was offered to 35–65% of patients with SVA [8,12]. In our population the most frequent indication for invasive treatment was intra-atrial reentrant tachycardia (80% of patients undergoing ablation), whereas in the previously described groups, the most common reason reported in 18.6–74.3% of all procedures was the atrioventricular reentrant tachycardia [2,6]. Catheter ablations of atrioventricular reentrant tachycardia in patients with EA are considered to be less effective than procedures targeting intra-atrial reentrant tachycardia [6,13]. This stands in line with our findings, in which the long-term effectiveness of atrioventricular reentrant tachycardia vs. intra-atrial reentrant tachycardia was 70% and 80%, respectively. In our report, the ultimate success rate of ablation, regardless of the specific type of SVA, is similar to the previously published outcomes. However, in our population, more than half of the subjects needed a re-do intervention in comparison to the rate of 0–33% reported in the previous studies [8,12,13]. The reasons for the higher rate of repeated procedures in our analysis are not clear. One possible factor is that the ablation technique itself and the experience of the operators evolved throughout the 20 years of follow-up and might not be directly comparable to the outcomes from recent years and over a shorter period of time. Another possible contributor is the varying rate of patients with a severe form of EA (in our study seen in almost 40% of individuals); unfortunately, these anatomical details are not reported in the previous studies [8,12,13].

In patients with normal cardiac anatomy, the success rate of catheter ablation of supraventricular arrhythmias is higher (for AVRT, 95–98%; for atrial flutter, 80–90%) than in similar procedures performed in patients with congenital heart defects [14,15,16]. The lower effectiveness of catheter ablation in the EA group is probably related to the location of the accessory pathways in the atrialized part of the right ventricle and the abnormal morphology of the endocardium, generating additional electrical potentials in this region [17]. Even though the efficacy of ablation in patients with EA is lower than in a structurally normal heart, it remains similar to that of other complex congenital heart defects [6,18,19,20]. The electroanatomical substrates for SVA in patients with congenital heart disease might vary a lot. In EA, right atrial enlargement is inherently present in all of the patients. Another contributor for arrhythmia in this population might also be fibrosis due to past surgery and long-term hemodynamic stress [21]. Apart from the hemodynamic overload of the right atrium, macro reentrant circuits also arise around the TV annulus through the abnormal cavotricuspid isthmus [19].

All the mentioned facts, as well as a small number of patients, could explain the lack of relationship between the occurrence of arrhythmias and the size of the heart chambers. Similarly to Hassan et al., we found no association between the parameters assessed in echocardiography or by CMR and the need for invasive treatment of supraventricular arrhythmia [13]. Since the values of parameters assessed by echocardiography and CMR in EA population are divergent, we performed a separate analysis for each imaging modality [10]. This outcome may hypothetically result from the fact that a significant part of the ablated group (66–80%) comprises patients with additional conduction pathways [2,6]. As this condition originates from the abnormal atrioventricular electric isolation during embryogenesis but not from the hemodynamic remodeling of the cardiac chambers due to anatomical defect of the tricuspid regurgitation [19].

The overall survival in adults with EA is satisfactory (95% to age 50 years, 82% to age 60 years, 72% to age 70 years, and 54% to age 80 years) and stands in line with the other data [22,23]. In our analysis, neither supraventricular arrhythmia nor ablation itself showed an effect on the survival in this population. This finding might seem surprising, as it is acknowledged that supraventricular arrhythmia rapidly conducted by atrioventricular node and/or accessory pathways may cause fast ventricular rhythm, resulting in a sudden decrease in cardiac output. As a consequence, the filling of the systemic ventricle decreases, and ultimately, it may cause sudden cardiac arrest. Nevertheless, the authors of observational studies published previously on this topic demonstrated contradictory conclusions. The impact of supraventricular arrhythmia on cardiac death during a 20-year follow-up was studied by Attie et al. on 72 unoperated EA patients, and the authors did not prove it to be statistically significant (HR = 1.13, 95% Cl 0.51–2.52) [24]. Similar observations among 73 adults with EA were presented by Crepin et al., who found that supraventricular arrhythmia did not affect survival in the described group, in contrast to age, associated malformations, functional class NYHA III and IV, cardiac failure, malaises, cyanosis, a raised cardiothoracic index, and a high mitro-tricuspid displacement indexed to body surface area [25]. In contrast to that, in the largest to date retrospective analysis of almost 1000 patients performed in Mayo Clinic, the univariable analysis proved supraventricular arrhythmia to be associated with the incidence of sudden cardiac death. Furthermore, the study by Rydman et al., analyzing 79 patients over 3.5 years, proved that a first-onset episode of atrial tachycardia increased the hazard ratio 11-fold for major adverse cardiovascular events (sustained ventricular arrhythmia/heart failure hospitalization/transplant/death) in the unrepaired EA population [26]. Interestingly, we did not report any case of malignant ventricular arrhythmia during a 20-year follow-up, although two patients who died suddenly could have been possibly accounted to this group. Nonetheless, the authors reported the SVA only in 20% of the study group, of which more than 40% had atrial fibrillation, while in our population, SVA was diagnosed in more than half of the patients, with atrial fibrillation present in 18% of them. Additionally, they showed that supraventricular arrhythmia due to accessory conduction pathways is not associated with major adverse cardiovascular events, and individuals with this arrhythmia were excluded from the final analysis. In our cohort, AVRT was a commonly diagnosed arrhythmia in the study group and we decided to include them into the study group. The reason for including those patients in our analysis is that any type of supraventricular arrhythmia, regardless of its origin within the atria, can be rapidly propagated to the ventricles and provoke a hemodynamic collapse, hence, sudden cardiac death. It should also be taken into account that almost half of our patients with SVA were treated with ablation and this could alter the conductive properties within atria with a potentially slower atrioventricular propagation of the electrical impulse. This could result in lower rate of ventricular response to SVA, and lower occurrence of the sudden death [2,22]. Having said that, it is worth noticing that we also did not show any impact of radiofrequency ablation on the survival of adults with Ebstein anomaly. Unfortunately, there are no data on this subject available in the previous reports.

Despite the broadly accepted thesis that supraventricular arrhythmia worsens the prognosis among adults with EA, there are a few publications with the contrary results. This may be a result of the statistically underpowered study groups. The second explanation for these contradictory outcomes is that the inclusion criteria in terms of type of arrhythmia were not consistent. Some authors made a clear distinction between AVRT and IART as having utterly different pathomechanisms; however, in our opinion, a potential hemodynamic collapse is mostly the question of rapid impulse propagation to the ventricle regardless of the type of SVA. Having said that, we believe that a particular interest should be put into a prospective analysis to elucidate the factors impacting survival in adults with EA, especially in the context of these questionable retrospective results coming from various papers.

## 5. Limitations

As it is a retrospective analysis, this report lacks the detailed catheter ablation data, and the follow-up is not structured. Additionally, it is a single-center study; thus, the group we examined is small, and each additional procedure impacts the final success rate. Nevertheless, the study size is comparable to the majority of the previously published reports and typical for congenital heart diseases.

## 6. Conclusions

Supraventricular arrhythmia is frequent in adults with Ebstein anomaly. Patients often require more than one catheter ablation but eventually are likely to be free from arrhythmias. The imaging parameters assessed by echocardiography or cardiac magnetic resonance do not seem to be associated with ablation outcomes. The impact of supraventricular arrhythmia itself or treatment with radiofrequency ablation is questionable and should be further investigated in this population.

## Figures and Tables

**Table 1:** Baseline population characteristics of the study group.

Variables	Study Group (*n* = 71)	Patients with SVA (*n* = 40)	Patients Requiring Ablation of SVA (*n* = 18)
Age, yrs	48.8 (±16.5)	49.5 (±15)	35.6 (±17.8)
Female (%)	31 (43.6%)	18 (45%)	13 (72.3%)
RBBB	64 (90.1%)	33 (82.5%)	17 (94.4%)
Mean QRS (ms)	137	122	135.6
Pacemaker	15 (21.1%)	9 (22.5%)	4 (22.2%)
Atrial fibrillation	7 (9.9%)	7 (17.5%)	1 (5.6%)
IART	26 (36.6%)	26 (65.5%)	14 (77.8%)
AVRT	14 (19.7%)	14 (35.0%)	9 (50%)
Comorbidities:			
Hypo/hyperthyroidism	8 (11.3%)	5 (12.5%)	3 (16.7%)
Hypertension	19 (26.8%)	5 (12.5%)	2 (11.1%)
Ischemic Heart Disease	3 (4.2%)	2 (5%)	1 (5.6%)
History of stroke	6 (8.5%)	3 (7.5%)	2 (11.1%)
Peripheral thromboembolism	4 (5.6%)	4 (10%)	1 (5.6%)
Medications:			
Antiplatelet drugs	12 (16.9%)	10 (25%)	1 (5.6%)
Anticoagulants	18 (25.4%)	16 (40%)	8 (44.4%)
Diuretics	25 (35.2%)	17 (42.5%)	7 (38.9%)
Beta Blockers	39 (54.9%)	27 (67.5%)	11 (61.1%)
Amiodarone	7 (9.9%)	5 (12.5%)	4 (22.2%)
Digoxin	3 (4.2%)	3 (7.5%)	0 (0%)

AF—atrial fibrillation; AVRT—atrioventricular reentrant tachycardia; IART—intra-atrial reentrant tachycardia; RBBB—right bundle branch block.

**Table 2:** Baseline population characteristics of patients with SVA not requiring ablation vs. requiring ablation.

Variables	Patients with SVA Not Requiring Ablation (*n* = 22)	Patients Requiring Ablation of SVA (*n* = 18)	*p*-Value
Age, yrs	56.8 (±13.9)	35.6 (±17.8)	*p* = 0.07
Female (%)	6 (27.3%)	13 (72.3%)	*p* = 0.004
RBBB	16 (72.7%)	17 (94.4%)	NS
Mean QRS (ms)	130.8	135.6	NS
Pacemaker	5 (22.7%)	4 (22.2%)	NS
Atrial fibrillation	6 (27.3%)	1 (5.6%)	NS
IART	12 (54.5%)	14 (77.8%)	NS
AVRT	5 (22.7%)	9 (50%)	NS

AF—atrial fibrillation; AVRT—atrioventricular reentrant tachycardia; IART—intra-atrial reentrant tachycardia; RBBB—right bundle branch block; NS—Non significiant.

**Table 3:** Basic echocardiographic parameters in the whole study group and in two subgroups (ablated vs. non-ablated) of patients with Ebstein anomaly.

	All Patients (*n* = 71)	Patients after Ablation (*n* = 18)	Patients Not Requiring Ablation (*n* = 53)	*p* Value Ablated vs. Non Ablated
LV (mm)	38.5 (±5.4)	37.4 (±5.9)	40.0 (±4.7)	*p* = 0.06
RV (mm)	56.4 (±15.8)	55.5 (±16.5)	55.3 (±13.9)	*p* = 0.9
RV/LV ratio	1.5 (±0.5)	1.5 (±0.5)	1.4 (±0.4)	*p* = 0.4
Cel index	1 (±0.45)	0.9 (±0.3)	0.96 (±0.6)	*p* = 0.9

**Cel index**—Celermajer index; **LV**—left ventricle; **RV**—right ventricle.

**Table 4:** Cardiac magnetic resonance parameters in patients with Ebstein anomaly and in two subgroups (ablated vs. non-ablated).

	All Patients (*n* = 45)	Ablated Patients (*n* = 10)	Non Ablated Patients (*n* = 35)	Ablated vs. Non-Ablated *p*-Value
TV Displ ind (mm/m^2^)	18.7 (±7.9)	17.4 (±10.5)	17.7 (±7.2)	*p* = 0.9
EDV LV ind (mL/m^2^)	64.7 (±16.7)	68.7 (±13.7)	70.9 (±21)	*p* = 0.9
SV LV ind (mL/m^2^)	38.4 (±12.4)	40.9 (±8.9)	41.5 (±15.8)	*p* = 0.65
LV EF (%)	56.9 (±11)	59.3 (±5.4)	57.2 (±9)	*p* = 0.5
EDV fRV ind (mL/m^2^)	134.9 (±63)	119.2 (±38.9)	148.5 (±69.3)	*p* = 0.2
SV fRV ind (mL/m^2^)	24.7 (±12.9)	24.8 (±10.2)	32.6 (±23,3)	*p* = 0.7
fRV EF (%)	40.3 (±12.9)	42.6 (±13.7)	41 (±11.4)	*p* = 0.7
RA + aRV (mL/m^2^)	15 (±9.5)	13.3 (±5.6)	12.7 (±5.1)	*p* = 0.3
fRV (mL/m^2^)	32.3 (±13.6)	37.1 (±19.9)	30,8 (±11.9)	*p* = 0.2
LA + LV (mL/m^2^)	10.2 (±3.3)	7.8(±3.3)	7.9 (±3.4)	*p* = 0.7
Cel index	0.75 (±0.45)	0.6 (±0.2)	0.8 (±0.5)	*p* = 0.3

**aRV**—atrialized right ventricle; **Cel index**—Celermajer index; **EDV ind**—end-diastolic volume index; **EF**—ejection fraction; **fRV**—functional right ventricle; **LGE**—late gadolinium enhancement; **LA**—left atrium; **LV**—left ventricle; **RA**—right atrium; **RV**—right ventricle; **SV ind**—systolic volume index; **TV Displ ind**—tricuspid valve displacement index.

## Data Availability

No new data were created or analyzed in this study. Data sharing is not applicable to this article.

## References

[1] Jost C.H.A., Connolly H.M., Dearani J.A., Edwards W.D., Danielson G.K. (2007). Ebstein’s anomaly. Circulation.

[2] Jost C.H.A., Tan N.Y., Hassan A., Vargas E.R., Hodge D.O., Dearani J.A., Connolly H., Asirvatham S.J., McLeod C.J. (2018). Sudden death in patients with Ebstein anomaly. Eur. Heart J..

[3] Baumgartner H., De Backer J., Babu-Narayan S.V., Budts W., Chessa M., Diller G.-P., Lung B., Kluin J., Lang I.M., Meijboom F. (2021). 2020 ESC Guidelines for the management of adult congenital heart disease: The Task Force for the management of adult congenital heart disease of the European Society of Cardiology (ESC). Eur. Heart J..

[4] Possner M., Gensini F.J., Mauchley D.C., Krieger E.V., Steinberg Z.L. (2020). Ebstein’s Anomaly of the Tricuspid Valve: An Overview of Pathology and Management. Curr. Cardiol. Rep..

[5] Hernandez-Suarez D.F., Kim Y., Villablanca P., Gupta T., Wiley J., Nieves-Rodriguez B.G., Rodriguez-Maldonado J., Maldonado R.F., Sant’Ana I.d.L., Sanina C. (2019). Machine Learning Prediction Models for In-Hospital Mortality After Transcatheter Aortic Valve Replacement. JACC Cardiovasc. Interv..

[6] Roten L., Lukac P., DE Groot N., Nielsen J.C., Szili-Torok T., Jensen H.K., Zimmermann M., Delacrétaz E. (2011). Catheter ablation of arrhythmias in ebstein’s anomaly: A multicenter study. J. Cardiovasc. Electrophysiol..

[7] Frescura C., Angelini A., Daliento L., Thiene G. (2000). Morphological aspects of Ebstein’s anomaly in adults. Thorac. Cardiovasc. Surg..

[8] Khositseth A., Danielson G.K., Dearani J.A., Munger T.M., Porter C.J. (2004). Supraventricular tachyarrhythmias in Ebstein anomaly: Management and outcome. J. Thorac. Cardiovasc. Surg..

[9] Lang R.M., Badano L.P., Mor-Avi V., Afilalo J., Armstrong A., Ernande L., Flachskampf F.A., Foster E., Goldstein S.A., Kuznetsova T. (2015). Recommendations for cardiac chamber quantification by echocardiography in adults: An update from the American Society of Echocardiography and the European Association of Cardiovascular Imaging. J. Am. Soc. Echocardiogr. Off. Publ. Am. Soc. Echocardiogr.

[10] Cieplucha A., Trojnarska O., Bartczak-Rutkowska A., Kociemba A., Rajewska-Tabor J., Kramer L., Pyda M. (2019). Severity Scores for Ebstein Anomaly: Credibility and Usefulness of Echocardiographic vs Magnetic Resonance Assessments of the Celermajer Index. Can. J. Cardiol..

[11] Kawel-Boehm N., Maceira A., Valsangiacomo-Buechel E.R., Vogel-Claussen J., Turkbey E.B., Williams R., Plein S., Tee M., Eng J., Bluemke D.A. (2015). Normal values for cardiovascular magnetic resonance in adults and children. J. Cardiovasc. Magn. Reson. Off. J. Soc. Cardiovasc. Magn. Reson..

[12] Legius B., Van De Bruaene A., Van Deyk K., Gewillig M., Troost E., Meyns B., Budts W. (2010). Behavior of Ebstein’s anomaly: Single-center experience and midterm follow-up. Cardiology.

[13] Hassan A., Tan N.Y., Aung H., Connolly H.M., Hodge D.O., Vargas E.R., Cannon B.C., Packer D.L., Asirvatham S.J., McLeod C.J. (2018). Outcomes of atrial arrhythmia radiofrequency catheter ablation in patients with Ebstein’s anomaly. EP Eur..

[14] Blomstrom-Lundqvist C., Scheinman M.M., Aliot E.M., Alpert J.S., Calkins H., Camm A.J., Campbell W.B., Haines D.E., Kuck K.H., Committee Members (2003). ACC/AHA/ESC Guidelines for the Management of Patients With Supraventricular Arrhythmias*—Executive Summary. Circulation.

[15] Spector P., Reynolds M.R., Calkins H., Sondhi M., Xu Y., Martin A., Williams C.J., Sledge I. (2009). Meta-analysis of ablation of atrial flutter and supraventricular tachycardia. Am. J. Cardiol..

[16] Gallego A.M., Díaz-Infante E., García-Bolao I. (2011). Spanish catheter ablation registry. 10th official report of the Spanish Society of Cardiology Working Group on Electrophysiology and Arrhythmias (2010). Rev. Esp. Cardiol..

[17] Cappato R., Schluter M., Weiß C., Antz M., Koschyk D.H., Hofmann T., Kuck K.H. (1996). Radiofrequency current catheter ablation of accessory atrioventricular pathways in Ebstein’s anomaly’. Circulation.

[18] Combes N., Derval N., Hascoët S., Zhao A., Amet D., Le Bloa M., Maltret A., Heitz F., Thambo J.-B., Marijon E. (2017). Ablation of supraventricular arrhythmias in adult congenital heart disease: A contemporary review. Arch. Cardiovasc. Dis..

[19] Sherwin E.D., Triedman J.K., Walsh E.P. (2013). Update on interventional electrophysiology in congenital heart disease: Evolving solutions for complex hearts. Circ. Arrhythm. Electrophysiol..

[20] Kumar S., Tedrow U.B., Triedman J.K. (2015). Arrhythmias in Adult Congenital Heart Disease: Diagnosis and Management. Cardiol. Clin..

[21] Walsh E.P. (2018). Ebstein’s Anomaly of the Tricuspid Valve: A Natural Laboratory for Re-Entrant Tachycardias. JACC Clin. Electrophysiol..

[22] Luu Q., Choudhary P., Jackson D., Canniffe C., McGuire M., Chard R., Celermajer D.S. (2015). Ebstein’s anomaly in those surviving to adult life—A single centre experience. Heart Lung Circ..

[23] Chang B.-C., Lim S.-H., Yi G., Hong Y.S., Lee S., Yoo K.-J., Kang M.S., Cho B.K. (2006). Long-term clinical results of tricuspid valve replacement. Ann. Thorac. Surg..

[24] Attie F., Rosas M., Rijlaarsdam M., Buendia A., Zabal C., Kuri J., Granados N. (2000). The adult patient with Ebstein anomaly. Outcome in 72 unoperated patients. Medicine.

[25] Crépin D., Jimenez M., Thambo J.B., Girardot R., Choussat A. (2004). Factors predictive of mortality in Ebstein’s anomaly. Arch. Mal. Coeur Vaiss..

[26] Rydman R., Shiina Y., Diller G.-P., Niwa K., Li W., Uemura H., Uebing A., Barbero U., Bouzas B., Ernst S. (2018). Major adverse events and atrial tachycardia in Ebstein’s anomaly predicted by cardiovascular magnetic resonance. Heart Br. Card. Soc..

